# Functional status after 3 years in ICU patients with traumatic brain injury

**DOI:** 10.1186/cc13666

**Published:** 2014-03-17

**Authors:** M Delange Van der Kroft, M Arias-Verdú, E Banderas-Bravo, E Curiel-Balsera, A Muñoz-Lopez, J Fernandez-Ortega, R Rivera-Fernandez, M Prieto-Palomino

**Affiliations:** 1 County Hospital of Axarquia, Velez Malaga, Spain; 2 Carlos Haya University Hospital, Malaga, Spain

## Introduction

The aim was to study the functional status at 1 year and after 3 years in ICU patients with traumatic brain injury (TBI).

## Methods

A prospective cohort study of patients with TBI admitted to Carlos Haya Hospital, Malaga, between 2004 and 2008.

## Results

We studied 531 patients, age 40.35 ± 19.75 years, APACHE II 17.94 ± 6.97, GCS admission 7.53 ± 3.83 points. Cranial tomography at admission was: diffuse injury type I (10.4%), diffuse injury type II (28.1%), diffuse injury type III (24.5%), diffuse injury type IV (8.3%), evacuated mass lesion (22.6%), non-evacuated mass lesion (6.2%). Hospital mortality was 28.6%. One-year mortality was 171 (32.2%) (missing: 6.6%). After 3 years, mortality was 181(34.1%) (16.2% missing). We followed 496 patients for 1 year after admission (35 were missing) and assessed their functional status using the GOS. Thus, 22.7% were normal and 20% presented some limitations but were self-sufficient, representing 55.6% of those who survived hospital admission. A total of 57.3% presented bad evolution (died: 34.5%, vegetative: 3%, disabled not self-sufficient: 19.8%). After 3 years we followed 445 patients and 86 (16.2%) were missing. Thus, 132 patients (29.7% of 445 patients) were normal and 15.3% presented some limitations but were self-sufficient, representing 75.76% of those who survived hospital admission. Fifty-six percent of 445 patients presented bad evolution (died: 40.7%, vegetative: 2.2%, disabled not self-sufficient: 12.1%). Of 445 patients studied after 3 years of admission 171 died, and of the 274 who survived 198 (72.2%) were in a similar functional situation after 1 year, 11 (4.01%) had worsened functional situation, of which 10 had died, and 65 (27.72%) had improved situation (*P *< 0.001).

## Conclusion

This study shows that approximately 75% of the patients who survived after 3 years from admission to the ICU with traumatic brain injury presented good recovery with functional situation normal or with some limitations but self-sufficient. Between 1 and 3 years after admission, approximately 25% of patients improved their functional situation.

